# Ultrasound-Guided Minimally Invasive Tissue Sampling: A Minimally Invasive Autopsy Strategy During the COVID-19 Pandemic in Brazil, 2020

**DOI:** 10.1093/cid/ciab885

**Published:** 2021-12-15

**Authors:** Amaro Nunes Duarte-Neto, Luiz Fernando Ferraz da Silva, Renata Aparecida de Almeida Monteiro, Jair Theodoro Filho, Thabata Larissa Luciano Ferreira Leite, Catia Sales de Moura, Michele Soares Gomes-Gouvêa, João Renato Rebellho Pinho, Cristina Takami Kanamura, Ellen Pierre de Oliveria, Kely Cristina Soares Bispo, Cássia Arruda, Aline Brito dos Santos, Flavia Cristina Gonçalves Aquino, Elia Garcia Caldini, Thais Mauad, Paulo Hilário Nascimento Saldiva, Marisa Dolhnikoff

**Affiliations:** 1 Brazilian Image Autopsy Study Group, Departamento de Patologia, Laboratório de Investigação Médica 05, Faculdade de Medicina da Universidade de São Paulo, São Paulo, Brazil; 2 Instituto Adolfo Lutz, São Paulo, Brazil; 3 Serviço de Verificação de Óbitos da Capital, Universidade de São Paulo, São Paulo, Brazil; 4 Departamento de Gastroenterologia, Laboratório de Investigação Médica 07, São Paulo, Brazil; 5 Departamento de Cardiopneumologia, Instituto do Coração, São Paulo, Brazil; 6 Departamento de Patologia, Laboratório de Investigação Médica 59, São Paulo, Brazil

**Keywords:** minimally invasive autopsy, COVID-19, pathology, ultrasound guided biopsy, minimally invasive tissue sampling, autopsy safety

## Abstract

**Background:**

Minimally invasive autopsies, also known as minimally invasive tissue sampling (MITS), have proven to be an alternative to complete diagnostic autopsies (CDAs) in places or situations where this procedure cannot be performed. During the coronavirus disease 2019 (COVID-19) pandemic, CDAs were suspended by March 2020 in Brazil to reduce biohazard. To contribute to the understanding of COVID-19 pathology, we have conducted ultrasound (US)–guided MITS as a strategy.

**Methods:**

This case series study includes 80 autopsies performed in patients with COVID-19 confirmed by laboratorial tests. Different organs were sampled using a standardized MITS protocol. Tissues were submitted to histopathological analysis as well as immunohistochemical and molecular analysis and electron microscopy in selected cases.

**Results:**

US-guided MITS proved to be a safe and highly accurate procedure; none of the personnel were infected, and accuracy ranged from 69.1% for kidney, up to 90.1% for lungs, and reaching 98.7% and 97.5% for liver and heart, respectively. US-guided MITS provided a systemic view of the disease, describing the most common pathological findings and identifying viral and other infectious agents using ancillary techniques, and also allowed COVID-19 diagnosis confirmation in 5% of the cases that were negative in premortem and postmortem nasopharyngeal/oropharyngeal swab real-time reverse-transcription polymerase chain reaction.

**Conclusions:**

Our data showed that US-guided MITS has the capacity similar to CDA not only to identify but also to characterize emergent diseases.

Coronavirus disease 2019 (COVID-19) was first diagnosed in Brazil on 25 February 2020, and the first death was recorded on 17 March 2020. By early June 2021, the COVID-19 epidemic in Brazil remains far from reaching control. Several difficulties and challenges to deal with the pandemics [1] resulted in >17 million confirmed cases and >477000 deaths so far [2]. The State of São Paulo is the most affected region, with the largest number of cases (3.3 million) and deaths (115000) [2]. Postmortem minimally invasive tissue sampling (MITS) is a well-recognized strategy for studying the epidemiology and pathophysiology of diseases. It is considerably less invasive than complete diagnostic autopsy (CDA) as a source of biological samples [3]. Minimally invasive autopsy, also known as MITS, includes molecular and histopathological examination and integration with image examinations and clinical data, and has proven to be an alternative to CDA in places or situations where CDA cannot be performed [3]. MITS blinded or guided by different image guiding technologies has been increasingly used in very different settings, depending on the type of death, centers’ imaging capacity, and local medical and public health capacities. Imaging guidance might expand MITS accuracy and the possibilities of targeting different organs [4–8].

MITS has been long recognized as a technique that could be a substitute for CDAs in certain circumstances. In Brazil, for instance, this procedure was used in the mid-1930s when postmortem liver sampling by viscerotomy took place to keep track of yellow fever cases [9]. Curiously, 84 years later [10], we have conducted an ultrasound (US)–guided MITS study on yellow fever during the São Paulo 2018 epidemic, confirming the rapid efficacy of this method during outbreaks [8].

Before the pandemic, the coroner autopsy service at the University of São Paulo Medical School conducted >15000 CDAs of natural deaths per year, providing the opportunity to test the potential of US-guided MITS against the CDA [8, 11]. US allows visualization of the organs and tissue sampling orientation and has low cost and good portability, allowing MITS to be conducted also in the hospital setting, outside of the autopsy room. Because of the unavailability of autopsy rooms that comply with national and international agencies’ biosafety recommendations such as negative air pressure and Biosafety Level 3 autopsy rooms [12], CDAs were suspended by March 2020 in Brazil for the period of the COVID-19 pandemic. As an unique opportunity to contribute to the incipient knowledge of COVID-19 pathophysiology, we decided to focus on US-guided MITS, which presents less risk of aerosols emission compared with CDA, to provide this critical information.

Here, we present the results of 80 US-guided MITS conducted between March and December 2020 in São Paulo. We describe the technical accuracy in obtaining adequate tissues and the ability to confirm COVID-19 diagnosis by different laboratory methods, describe the pulmonary and systemic involvement of the disease, and present relevant data about the safety of MITS in COVID-19. We also show the contribution of US-guided MITS to important pathophysiological insights in the scenario of a novel disease.

## METHODS

This is an observational case series study based on the autopsies of patients who died of COVID-19 at Clinics Hospital, University of São Paulo School of Medicine (HC-FMUSP) in 2020, in São Paulo, Brazil. This protocol was approved by the HC-FMUSP Ethical Committee and Institutional Review Board (protocol number 3.951.904). From March to December 2020, 4633 patients with confirmed COVID-19 were hospitalized in our institution, with 1573 deaths (34% mortality rate). During this period, we performed US-guided MITS of patients who died with pre- or postmortem confirmation of COVID-19.

The inclusion criteria followed the World Health Organization (WHO) definition for COVID-19 confirmed cases, which is laboratory confirmation of severe acute respiratory syndrome coronavirus 2 (SARS-CoV-2) infection, regardless of clinical signs [13]. We considered a positive identification of SARS-CoV-2 infection when real-time reverse-transcription polymerase chain reaction (rRT-PCR) was positive in any of the following samples: pre- or postmortem nasopharyngeal/oropharyngeal swab (NP), or postmortem sampled tissues. rRT-PCR in NP (NP-PCR) for the detection of SARS-CoV-2 was performed during hospitalization. In case of absence of a premortem NP-PCR, a postmortem NP was collected immediately before MITS. Additionally, PCR of frozen lung tissue was performed in all deceased patients with suspected disease and without confirmation with the NP-PCR.

The cases also fulfilled the following requirements: ethical board approval, written consent from the next of kin, autopsies requested by the clinical staff, and MITS team availability. The procedures were performed at the HC-FMUSP Research Center Imaging Platform in the Autopsy Room (https://pisa.hc.fm.usp.br/).

Epidemiological, clinical, and laboratory data were obtained from medical charts. The short version of the WHO Verbal Autopsy Questionnaire was applied to the next of kin to complement the prehospital admission information.

### US-Guided MITS Protocol

US-guided MITS was performed using a standard safety protocol as previously described [14] Access to the autopsy room was limited to 2 people: the US examiner and the supporting technician. The personnel involved directly or indirectly with the MITS procedure were tested weekly for COVID-19 with NP-PCR [12, 14–16].

**Figure 1.**
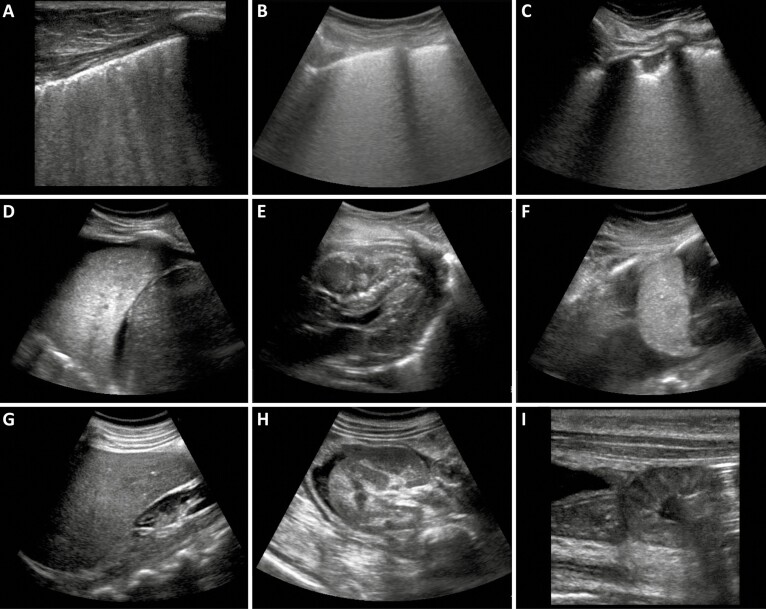
Representative postmortem ultrasound images during ultrasound-guided minimally invasive tissue sampling procedures. *A*, Lung of non–coronavirus disease 2019 (COVID-19) patient showing irregular pleural line and confluent B lines indicating edema. *B–D*, Lung of COVID-19 patient showing diffuse edema, known as white lung (*B*); peripheral consolidation and irregular pleural line (*C*); pulmonary consolidation, also known as pulmonary hepatization and pleural effusion (*D*). *E*, Heart of a COVID-19 patient showing hyperechogenic foci in interventricular septum, usually correlated to hypovascularized areas. *F*, Spleen of COVID-19 patient and ascites. *G*, Liver of COVID-19 patient showing echogenicity compatible with steatosis. *H*, Cross-section of a kidney and ascites. *I*, Thickened small bowel wall.

We used portable SonoSite M-TurboR (Fujifilm, Bothell, Washington) US equipment with C60x (5-2 MHz Convex) multifrequency broadband transducers and generation of Digital Imaging and Communications in Medicine standard images. The US images were used to localize and guide the sampling in several organs and to select the most affected areas within each organ. Tissue sampling was performed with Tru-Cut semi-automatic coaxial needles (14G; 20cm in length). Figure 1 shows representative US images from different organs.

The following tissue samples were collected under US guidance: lungs (8 samples from each lung), liver (2 samples), both kidneys (1 sample each), spleen (1 sample), and heart (1–3 samples). Other tissues were sampled without direct image guidance: skeletal muscle (femoral quadriceps), skin (left thigh, with a 5-mm punch), and brain (trans-sphenoidal puncture). Intestine, periodontal tissue, salivary glands, adipose tissue, bone marrow, and thyroid were collected in selected cases. Tissue samples for molecular tests were immediately frozen and stored at –80°C. Tissue samples for histopathological analysis were fixed in buffered 10% formalin and routinely processed. In selected cases, tissue samples were fixed in glutaraldehyde for ultrastructural analyses.

### Molecular and Immunohistochemical Detection of SARS-CoV-2

Immunohistochemistry (IHC) was performed on lung samples from selected cases to detect SARS-CoV-2 nucleocapsid protein. The criteria for case selection included cases with negative premortem and postmortem PCR and suggestive pulmonary histology; unusual cases (eg, children, puerperal women); and cases with particular pulmonary histological findings (eg, squamous metaplasia, pulmonary “pseudo-cysts”). A detailed description of the IHC protocol has been previously reported [16]. The primary antibody was a mouse monoclonal antibody (6H3; GeneTex, Irvine, California), at 1:500 dilution.

We employed PCR for RNA detection of SARS-CoV-2 in frozen lung tissue of all patients who did not have the diagnosis confirmation prior to death. Tissues were submitted to homogenization using the FastPrep™ instrument (MP Biomedical, São Caetano do Sul, Brazil), and nucleic acid extraction was performed using the TRIzol® reagent (Invitrogen, Carlsbad, California). Molecular detection of SARS-CoV-2 was performed as previously described [17–19]

### Ultrastructural Analyses

Pulmonary tissue samples from 7 patients were examined under transmission electron microscope (EM). Tissues were fixed in 2% glutaraldehyde, postfixed in 1% osmium tetroxide, stained in 1% aqueous uranyl acetate, and embedded in epoxy resin. Ultrathin sections were double-stained by uranyl acetate and lead citrate. Micrographs were obtained with a Jeol JEM 1010 EM (80kV; Tokyo, Japan).

### Diagnoses of Causes of Death by US-Guided MITS

To determine the immediate and underlying cause of death, a senior pathologist and an infectious disease specialist together considered the following information: (1) clinical information obtained with the next of kin and hospital records; (2) postmortem US information; (3) histopathological analysis; and (4) when available, pre- or postmortem NP-PCR, lung tissue PCR, SARS-CoV-2 IHC, and ultrastructural analysis. When there was any doubt about the final diagnosis, the case was discussed in a panel with 3 senior pathologists. All reported causes were coded by the São Paulo State Health Bureau according to the *International Classification of Diseases*, *11th Revision*.

### Developments in the Technique

**Figure 2.**
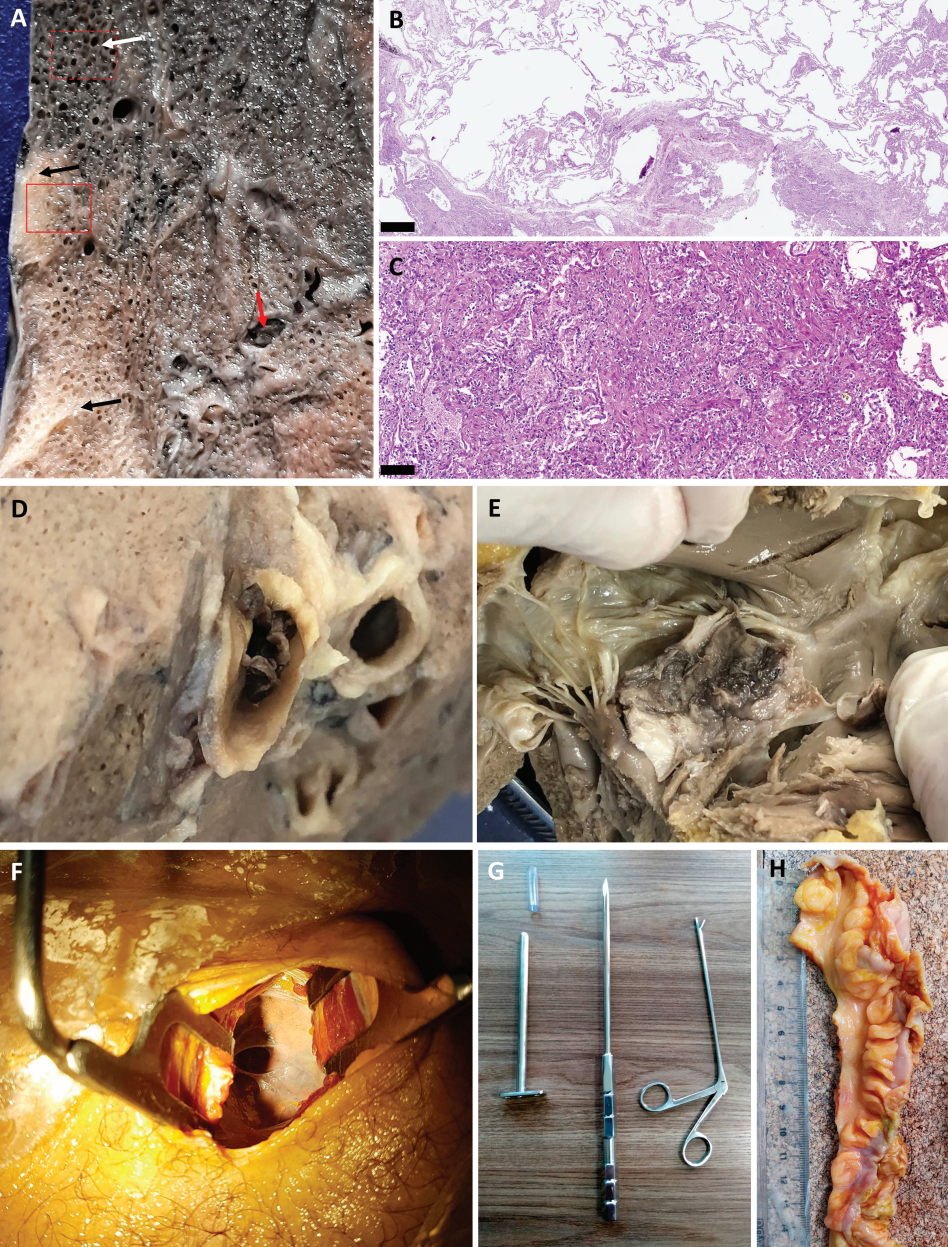
*A*, Macroscopic view of pulmonary fragment sampled from a late coronavirus disease 2019 case obtained by a small thoracotomy guided by ultrasound image. Red arrows indicate thromboembolic event. *B*, Microscopic view showing areas of overinflation and disrupted alveolar septa (corresponding to white arrow and red dotted line rectangle marked in *A*). *C*, Foci of subpleural condensation with fibrotic bundle deposition and reactive epithelium (corresponding to black arrows and solid red line rectangle marked in *A*). *D*, Macroscopic view of a pulmonary thrombus. *E*, Tricuspid valve leaflet of the same patient showing a valvular thrombus. *F*, Skin and chest wall incision. *G*. Instruments used to insert optics and forceps for dissections. *H*, Segment of the intestines obtained with the dissection technique. Scale bar for microscopic images = 500 µm.

After 9 months of using US-guided MITS, the method was modified to obtain larger fragments than those obtained with the 14G needle, which was necessary to clarify the basic cause of death in specific situations, such as myocardial infarction or pulmonary thromboembolism involving large vessels. For this purpose, we performed scalpel dissections guided by the US, through small skin and chest wall incisions (up to 3.0cm) over the area of interest (mainly lungs and heart). We performed 3 additional cases using this technique (Figure 2).

### Ethical Considerations

This work was approved by the Clinics Hospital, University of São Paulo School of Medicine Ethical Committee (protocol number 3951.904).

## RESULTS

### MITS Safety

None of the 10 professionals involved directly or indirectly with the MITS procedure were infected with SARS-CoV-2 according to the weekly NP-PCR tests. The autopsy staff were vaccinated against COVID-19 in January 2021, according to the Brazilian national vaccination program.

### US-Guided MITS Portability

Fifteen US-guided MITS were carried out in other institutes of our medical complex to avoid the delay in transporting the bodies to the autopsy service. One procedure was performed inside the intensive care unit (ICU)—autopsy at the bedside—without negative pressure. In all these cases, the equipment for the MITS, including the portable US, was easily transported by the autopsy team.

### Patients and COVID-19 Diagnostic Results

We performed a total of 80 US-guided MITS on patients with COVID-19 in 2020. Only 1 family refused the procedure. Table 1 shows the demographics and clinical data of the 80 patients. Forty-one patients were male (51.3%), and the median age was 56 (Range: 0.6–88) years old. This case series includes 5 children (Range: 0.6–15 years old) [17] and 4 puerperal women. The time span between symptom onset and hospital admission had a median of 6.2 (range, 0–26) days; the disease had a median duration of 23.9 (range, 0–95) days, and 7 patients (8.75%) were hospitalized for <24 hours. Main comorbidities included systemic arterial hypertension (50%), overweight (34.3%), diabetes mellitus (28.8%), cardiomyopathy (26.3%), and obesity (18.6%). All patients had severe respiratory distress, and 68 (85.0%) died because of refractory hypoxemia. Twelve patients (15%) with positive PCR results died of other immediate causes, including cardiogenic shock (4 cases), multisystem inflammatory syndrome in children (MIS-C) (3 cases) [17], pulmonary embolism (2 cases), ischemic stroke (1 case), hemorrhagic stroke (1 case), and pneumothorax (1 case). All patients underwent mechanical ventilation and received vasoactive drugs and broad-spectrum antibiotics.

**Table 1.** Demographics, Disease Intervals, Diagnostics, and Comorbidities of 80 Adult Deaths From Coronavirus Disease 2019 in São Paulo, Brazil

Demographics and Disease Intervals	No. (%)^a^
Age, y, median (range)	56 (0.6–88)
Sex	
Female	39 (48.8)
Male	41 (51.3)
Symptoms to hospital admission interval, d, mean ± SD (range)	6.2 ± 4.7 (0–26)
Symptoms to death interval, d, mean ± SD (range)	23.9 ± 16.1 (0–95)
Hospital admission to death interval, d, mean ± SD (range)	17.7 ± 15.8 (0–92)
ICU admission to death interval, d, mean ± SD (range)	13.1 ± 13.4 (0–86)
COVID-19 diagnosis	
Premortem diagnosis	50 (62.5)
Postmortem diagnosis	30 (27.5)
Strategy for postmortem diagnosis	
Viral identification in NOPS RT-PCR only	10 (33.3)
Viral identification in tissue samples only	15 (50.0)
Viral identification in both NOPS RT-PCR and tissue samples	5 (16.7)
Tool combinations for postmortem diagnosis	
NOPS RT-PCR only	10 (33.3)
IHC only	7 (23.3)
Tissue RT-PCR only	1 (3.3)
NOPS RT-PCR + IHC	3 (10.3)
NOPS RT-PCR + lung tissue RT-PCR	1 (3.3)
NOPS RT-PCR + IHC + tissue RT-PCR + EM	1 (3.3)
IHC + tissue RT-PCR	4 (13.3)
IHC + tissue RT-PCR + EM	2 (6.7)
IHC + EM	1 (3.3)
Comorbidities	
Previous diseases, No./Total No. (%)	
Arterial hypertension	40/80 (50.0)
Overweight	24/70 (34.3)
Diabetes mellitus	23/80 (28.8)
Ischemic cardiopathy	21/80 (26.3)
Obesity	13/70 (18.6)
Immunosuppression	14/80 (17.5)
Vascular disease	11/80 (13.8)
Neoplasia	10/80 (12.5)
Chronic renal disease	6/80 (7.5)
COPD	5/80 (6.3)
Asthma	4/80 (5.0)
HIV	1/80 (1.3)
Smoking history	
Nonsmoker	59 (73.8)
Smoker	9 (11.3)
Ex-smoker	12 (15.0)

Data are presented as No. (%) unless otherwise indicated.

Abbreviations: COPD, chronic obstructive pulmonary disease; COVID-19, coronavirus disease 2019; EM, electron microscopy; HIV, human immunodeficiency virus; ICU, intensive care unit; IHC, immunohistochemistry; NOPS, nasopharyngeal/oropharyngeal swab; RT-PCR, reverse-transcription polymerase chain reaction; SD, standard deviation.

Five patients (6.3%) were children (Range: 0.6–15 years old). A detailed description of these cases is available in a different publication [17]. Three children died with MIS-C secondary to COVID-19 as the underlying cause of death; the other 2 had different causes of death as follows: 1 with Edwards syndrome followed by SARS-CoV-2 pneumonia, and 1 with adrenal carcinoma followed by a combination of bacterial and COVID-19 pneumonia and disseminated thrombosis.

Fifty patients had an antemortem diagnosis of COVID-19 by NP-PCR; 30 patients had only a postmortem diagnosis of COVID-19. Among these, 10 (33.3%) were diagnosed using postmortem NP-PCR; 15 (50%) were diagnosed by viral detection in lung tissue samples (tissue PCR, IHC, or EM). In another 5 cases (16.7%), both strategies were used. At least 4 patients with suspected COVID-19 (5% of total) needed tissue sampling for the final diagnosis (considering only those with negative pre- and postmortem nasopharyngeal/oropharyngeal swab).

### Tissue Sampling Representativeness and Accuracy

Table 2 shows the number of patients biopsied in each organ and the accuracy of the MITS biopsies per organ. For the lungs, the samples were accurate and representative in 81.7% to 90.1%, depending on the lung region. The set of pulmonary biopsies was accurate in 84% of the cases, and all patients had at least 1 representative pulmonary sample. For the other organs, the most accurate sampling was achieved in liver (98.7%) and heart (97.5%).

**Table 2.** Representability of Ultrasound-Guided Minimally Invasive Tissue Sampling, by Organ

Organ/Site	Cases Sampled	Organ Accuracy, %
Lung^a^		
RL-SM	71/80	80.5
RL-IM	70/80	87.3
RL-SL	69/80	82.8
RL-IL	78/80	83.5
LL-SM	77/80	87.0
LL-IM	71/80	88.9
LL-SL	66/80	86.4
LL-IL	64/80	89.1
All areas	80/80	85.6
Heart^a^	80/80	97.5
Liver^a^	75/80	98.7
Kidney^a^		
Right kidney	75/80	71.3
Left kidney	71/80	71.8
All areas	76/80	69.1
Spleen^a^	67/80	90.3
Skin^b^	62/80	98.6
Brain^c^	64/80	100.0

Abbreviations: LL-IL, left lung inferior and lateral; LL-IM, left lung inferior and medial; LL-SL, left lung, superior and lateral; LL-SM, left lung, superior and medial; RL-IL, right lung inferior and lateral; RL-IM, right lung inferior and medial; RL-SL, right lung, superior and lateral; RL-SM, right lung, superior and medial.

a Ultrasound-guided sampling with 14G semi-automatic biopsy system.

b Direct dissection/punch.

c Transethmoidal puncture.

### Pulmonary Histological Changes

**Figure 3.**
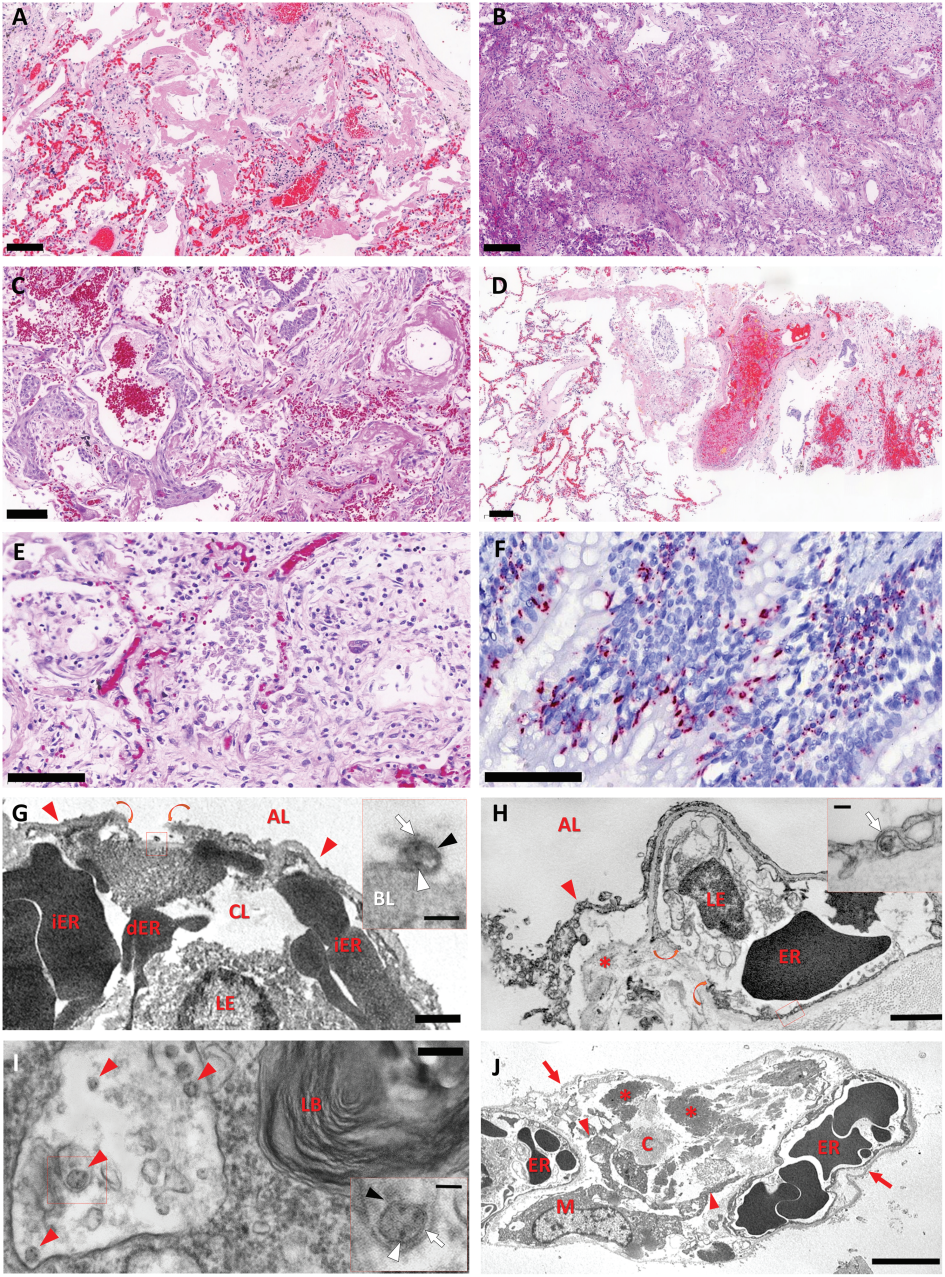
Pulmonary histological findings of 80 fatal cases of coronavirus disease 2019, via ultrasound-guided minimally invasive tissue sampling. *A–F*, Scale bars = 100 µm. *A*, Exudative diffuse alveolar damage with hyaline membranes in the alveolar space, alveolar edema, and congestion. Hematoxylin and eosin (H&E) staining. *B*, Proliferative diffuse alveolar damage. H&E staining. *C*, Squamous metaplasia of the alveolar epithelium. H&E staining. *D*, Pulmonary artery with thrombus. H&E staining. *E*, Area of fibroproliferative septal damage exhibiting alveolar cells with type 2 pneumocyte hyperplasia, with enlarged and multinucleated cells. H&E staining. *F*, Positive immunostaining, detecting severe acute respiratory syndrome coronavirus 2N protein in the cytoplasm of ciliary bronchiolar cells and alveolar cells. *G*, Representative electron micrograph of interalveolar septum. Capillary lumen (CL) is filled with leukocytes (LE), intact erythrocytes (iER), and damaged erythrocytes (dER). Thin type I epithelial cells (arrowheads) face the alveolar lumen (AL). There is an area of disrupted type I epithelial cell (borders shown in curved arrows). A higher-magnification image of the boxed area (insert) shows extracellular virion attached to the denuded basal lamina (BL); note the viral envelope (black arrowhead), nucleocapsids (white arrowhead) and spikes (white arrow) at virus particle surface. Scale bar = 1 µm; inset scale bar = 100nm. *H*, Electron micrograph showing the detachment of epithelial layer (arrowhead)—that lined the AL— from the interstitium (asterisk), and the rupture (indicated by curved arrows) of capillary endothelial cells (LE: leucocyte; ER: erythrocytes). The inset shows the boxed area of the endothelial cell containing a coronavirus particle (arrow). Scale bar = 2 µm; inset scale bar = 100nm. *I*, Electron micrograph of cytoplasmic area of an alveolar type II pneumocyte, showing lamellar body (LB) and a membrane compartment with some viral particles (red arrowheads). The inset corresponds to a higher magnification of the virus particle enclosed by the box frame showing a membrane envelope (black arrowhead), black dots resembling nucleocapsids (white arrowhead) in the interior, and spikes at virus surface (white arrow). Scale bar = 200nm; inset scale bar = 100nm. *J*, Low-magnification electron micrograph of a thickened interalveolar septum showing an extensive denudation of alveolar epithelial basement membranes (arrow). The bulk of interstitium is filled with a myofibroblast (M), its extensions (arrowheads), and a great amount of collagen (C) and elastic (asterisks) fibers. Note that capillaries contain abnormal-shaped erythrocytes (ER) presenting either protrusion or indentation, adjusting one another. Scale bar = 5 µm.

Table 3 shows the main pulmonary and systemic changes in COVID-19: exudative diffuse alveolar damage (DAD) (97.5%), proliferative DAD (78.5%), vascular thrombosis in small vessels or capillaries (63.3%), and increased number of megakaryocytes (53.2%). Other patterns of acute lung injury, such as organizing pneumonia and acute fibrinous organizing pneumonia, were present in 13 cases (16.2%) and were associated with DAD. We also identified viral cytopathic changes in epithelial cells (100%), and interstitial lymphocytic infiltrate (n = 78 [98.8%]) (Figure 3A–F). EM demonstrated epithelial and endothelial destruction, collagen deposition, and viral particles in type I and type 2 pneumocytes and in endothelial cells (Figure 3G–J).

**Table 3.** Histopathological Findings in Deceased Coronavirus Disease 2019 Patients Submitted to Ultrasound-Guided Minimally Invasive Tissue Sampling

Pathological Finding	No. (%)
Lung (80 cases)	
Acute DAD	77 (97.5)
Mild	36 (45.6)
Intense	41 (51.9)
Proliferative DAD	62 (78.5)
Mild	26 (32.9)
Intense/pulmonary fibrosis	36 (45.6)
Lymphocytic infiltrate	78 (98.8)
Mild	62 (78.5)
Intense	16 (20.3)
Vascular thrombosis	50 (63.3)
Mild	20 (25.3)
Intense	30 (38.0)
Hyperplasia of type II pneumocytes	80 (100.0)
Mild	24 (30.4)
Intense	55 (69.6)
Viral atypia with giant cell	30 (37.9)
Mild	25 (31.6)
Intense	5 (6.3)
Reactive endothelium	47 (59.5)
Squamous metaplasia	27 (34.2)
Mild	10 (12.7)
Intense	17 (21.5)
Alveolar hemorrhage	54 (68.4)
Mild	36 (45.6)
Intense	18 (22.8)
Pneumonia pattern	69 (87.3)
Interstitial	26 (32.9)
Suppurative	43 (54.4)
Organizing pneumonia	13 (16.2)
OP^a^	10 (12.5)
AFOP^a^	6 (7.5)
Bacterial colonies	9 (11.4)
Fungus infection	1 (1.2)
Miliary tuberculosis	1 (1.2)
Increased number of megakaryocytes^b^	42 (53.2)
Angiomatosis	12 (15.4)
Cysts	10 (12.8)
Necrosis/infarction	7 (9)
Vasculitis	3 (3.8)
Liver (75 cases)	
Shock-related alterations	75 (100.0)
Centrilobular/midzonal necrosis	49 (65.3)
Steatosis	42 (56.0)
Sinusoidal neutrophils	36 (48.0)
Periportal infiltrate	31 (41.3)
Kupffer cell hypertrophy	22 (29.3)
Kupffer hemophagocytosis	7 (9.3)
Fibrin thrombi hepatic sinusoids	5 (6.6)
Steatohepatitis	3 (4.0)
Brain (72 cases)	
Astrogliosis	67 (93.1)
Edema-congestion	43 (59.7)
Small vessel disease	27 (37.5)
Hemorrhage	18 (25.0)
Massive	2 (3.0)
Focal	17 (22.0)
Satellitosis	16 (22.2)
Fibrin thrombi	9 (12.5)
Neuropil rarefaction	6 (8.3)
Perivascular inflammation	3 (4.2)
Lymphoid focal meningitis	3 (4.2)
Ischemia	2 (2.8)
Kidney (69 cases)	
Acute tubular necrosis	68 (98.6)
Hypertension alterations (vascular wall thickening + glomerular sclerosis)	40 (58.0)
Mesangial cell hyperplasia	37 (56.3)
Interstitial edema	32 (46.4)
Interstitial nephritis	23 (33.3)
Thrombosis	13 (18.8)
Kimmelstiel-Wilson lesion	10 (14.5)
Collapsing nephritis	9 (13.0)
Casts	4 (5.8)
Pyelonephritis	6 (8.7)
Heart (78 cases)	
Cardiomyocytes hypertrophy	63 (80.8)
Myocardial sclerosis	49 (62.8)
Edema	45 (57.7)
Acute ischemia	23 (29.5)
Myocarditis	15 (19.2)
Thrombosis	7 (9.0)
Spleen (61 cases)	
Lymphoid depletion	61 (100.0)
Red pulp hemorrhage	51 (83.6)
Splenitis	35 (57.4)
Extramedullary hematopoiesis	19 (31.1)
Red pulp ischemia	14 (23.0)
Fibrin thrombi	6 (10.0)
Hemophagocytosis	6 (9.8)
Bone marrow (19 cases)	
Cellularity	10 (52.7)
Hypocellular	9 (47.4)
Hypercellular	1 (5.3)
Reduced maturation	5 (26.3)
Emperipolesis	3 (15.8)
Skin (65 cases)	
Perivascular infiltrate	50 (76.9)
Periadnexal infiltrate	11 (16.9)
Thrombosis	9 (13.8)

Data are presented as No. (%). Mild: alterations observed, focally in 1 pulmonary sample; intense: alterations in >50% of the area of a sample, in >2 sampled areas on the same side, or bilaterally.

Abbreviations: AFOP, acute fibrinous and organizing pneumonia; DAD, diffuse alveolar damage; OP, organizing pneumonia.

a Three cases had both patterns.

b Increased number of pulmonary megakaryocytes: >2 cells in a microscopic field of ×200 magnification.

**Figure 4.**
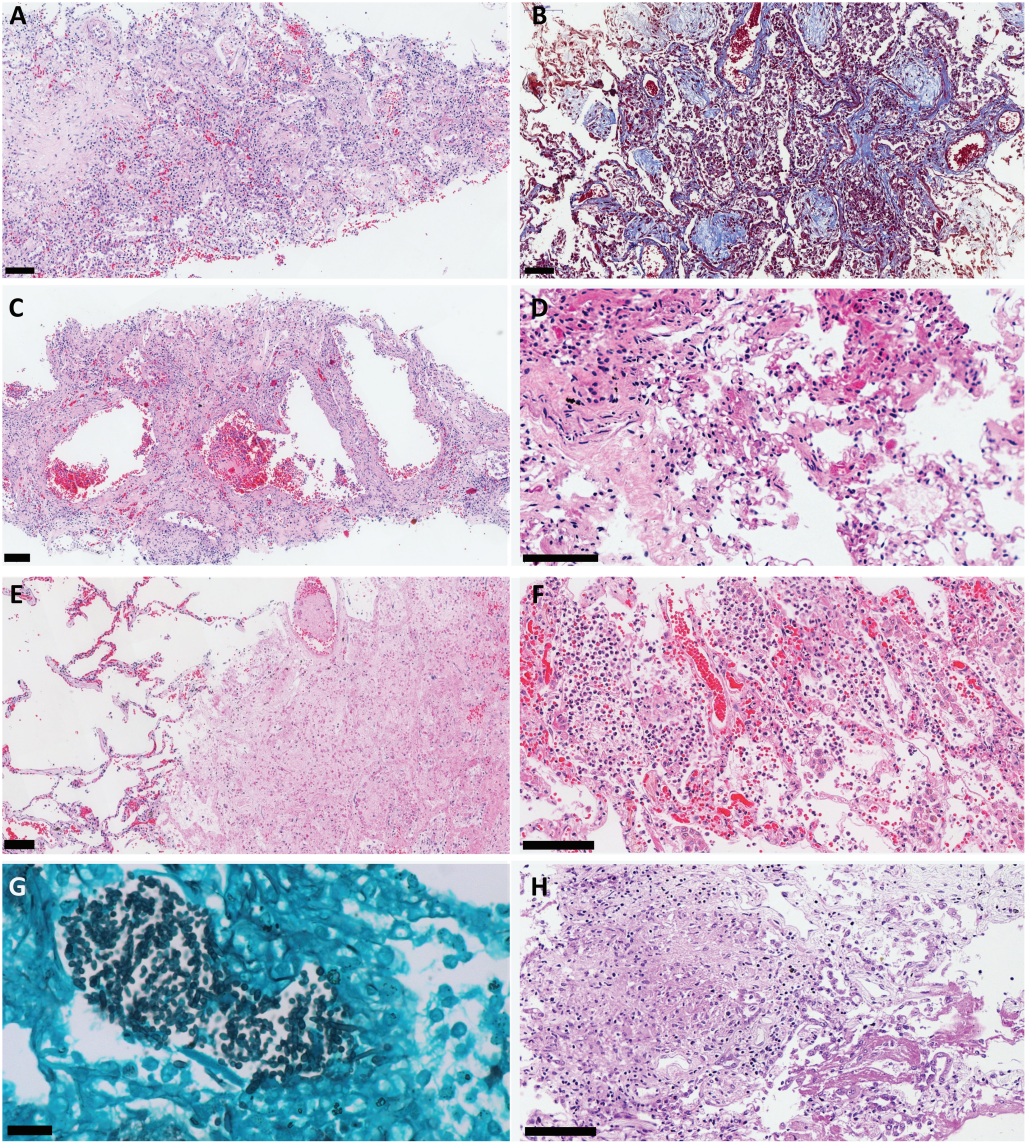
Pulmonary histological findings of 80 fatal cases of coronavirus disease 2019 (COVID-19), ultrasound-guided minimally invasive tissue sampling. All scale bars = 100 µm unless otherwise specified. *A*, Severe proliferative/organizing diffuse alveolar damage with lymphocytic septal infiltrate, and fibroblast intra-alveolar proliferation, depositing collagen matrix. Hematoxylin and eosin (H&E) staining. *B*, Collagen matrix deposition in alveolar spaces. Masson trichrome staining. *C*, Severe interstitial fibrosis with “pseudo-cystic” formations due to barotrauma. H&E staining. *D*, Septal capillaries with angiomatoid aspect associated with tiny fibrin clots. H&E staining. *E*, Thrombus in a pulmonary artery, with surrounding parenchyma exhibiting coagulative necrosis. H&E staining. *F*, Secondary suppurative bacterial pneumonia. H&E staining. *G*, Grocott silver stain showing hyphae compatible with *Candida* spp in a case of COVID-19 with bacterial pneumonia. Scale bar = 20 µm. *H*, Pulmonary miliary tuberculosis associated with COVID-19. H&E staining.

Suppurative pneumonia was observed in 43 cases (54.4%), and bacterial colonies (cocci or coccobacilli) were found in 9 of these (11.4%); *Candida* spp forms were observed in an additional case (Figure 3). Miliary tuberculosis was found in 1 case, affecting lungs, liver, and spleen. Particular pulmonary histological patterns were observed, such as pseudocystic formation due to interstitial fibrosis and barotrauma (n = 10 [12.8%]), angiomatoid pattern of septal capillaries (n = 12 [15.4%]), and coagulative necrosis/pulmonary infarction secondary to microthrombosis (n = 7 [9%]) (Figure 4). The IHC for SARS-CoV-2 was positive in 11 cases (13.8%). The positivity staining included cytoplasm of ciliary cells and alveolar epithelial cells as well as alveolar macrophages, in a granular pattern, sometimes in “dot-like” staining.

A semiquantitative analysis of some histological parameters is provided in Table 3.

### Extrapulmonary Findings

**Figure 5.**
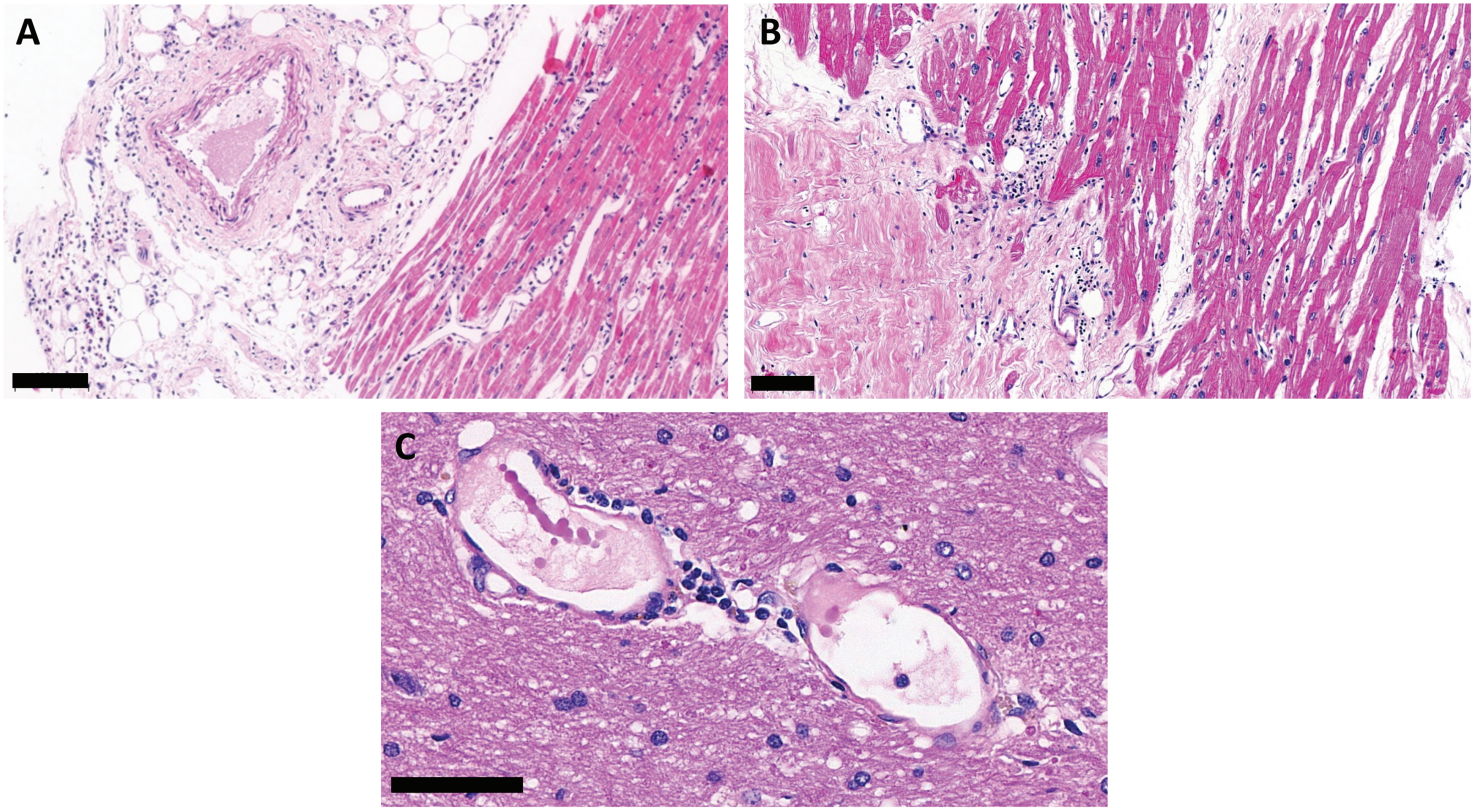
Extrapulmonary histological features of 80 fatal cases of coronavirus disease 2019, autopsied by the ultrasound-guided minimally invasive tissue sampling method. *A*, Epicarditis and myocarditis related to severe acute respiratory syndrome coronavirus 2 infection. *B*, Discrete interstitial lymphocytic myocarditis. Scale bar = 100 µm. *C*, Cerebral vessel exhibiting dilation, with perivascular mononuclear infiltrate, hemosiderin deposition, and tiny fibrin thrombi. Scale bar = 50 µm.

The most common extrapulmonary changes attributed to comorbidities included cardiomyocyte hypertrophy (80.8%) and myocardial fibrosis (62.8%), hypertensive nephropathy (58%), liver steatosis (56%), and cerebral small-vessel disease (37.5%). The most frequent alterations due to shock were hepatic centrilobular congestion (100%) and centrilobular/midzonal necrosis (65.3%), acute tubular necrosis (98.6%), and cerebral edema and congestion (60%). Common inflammatory findings, attributed to the SARS-CoV-2 infection or systemic inflammation, were superficial dermatitis with moderate lymphocytic perivascular (76.9%) and Periadnexal (17%) infiltrate, splenitis (57.4%), glomerular mesangial cell hyperplasia (56.3%), mild lymphocytic interstitial myocarditis (19.2%), hemophagocytosis in spleen (9.8%) and in Kupffer cells (9.3%), and cerebral perivascular inflammatory infiltrate (4.2%) and lymphocytic meningitis (4.2%). Vascular alterations included fibrin thrombi in cerebral vessels (12.5%), hepatic sinusoids (6.6%), glomerular capillaries (18.8%), skin (13.8%), myocardial vessels (9%), and spleen (10%) (Figure 5).

## DISCUSSION

We present the results of the application of US-guided MITS during a year of the COVID-19 pandemic in Brazil, the epicenter of the disease in the world by this date. In a scenario where CDAs were interrupted by law combined with the urgent necessity to understand the pathology of a novel ominous disease, US-guided MITS proved to be feasible, safe, and fast, providing an adequate representation of all vital organs, including the lungs, the main target organ of the disease. This is the second time that our group used US-guided MITS during an epidemic [8]. The method was also shown to be effective outside the autopsy room, allowing some cases to be performed within the hospital setting, confirming its portability.

One of the important issues related to MITS concerns the representation of the different organs. Low representativeness can impair the evaluation of heterogeneous or localized diseases. Our success rate in collecting representative samples was high (70%–100%), and highest in the central nervous system, liver, and heart and lowest in the kidneys. The lung accuracy varied from 81% to 90% depending on the region. These results are superior (particularly for the kidneys) or comparable with those found by other groups that compared US-guided MITS with blind needle biopsies [4–7]. We were able to characterize the different histological components of COVID-19 severe pneumonia [14, 20] and to understand the systemic tissue alterations.

When compared with large studies on COVID-19 using CDAs, one can see that using US-guided MITS no major diagnoses, except for large vessel thrombosis, were missed [21–25]. With a large number of cases, our study confirms our previous observations [14] that the lungs are the main target of the disease, with SARS-CoV-2 pneumonia being mainly responsible for the progression to respiratory failure. These results were also similar to those reported early in the pandemic [26] and in more recent publications [27].

The predominant pulmonary change was DAD, with overlapping of the exudative and proliferative phases, with progression to pulmonary fibrosis. In another study, we were able to expand this information by showing that the histological progression of the pulmonary lesion seems to be associated with age, body mass index, and disease duration [20]. This is also consistent with other series that showed similar patterns of evolution of disease according to severity and time [28].

Other histological patterns of acute lung injury were also observed, such as acute fibrinous organizing pneumonia and organizing pneumonia, which are important to be recognized for possible corticosteroid therapy [29–31]; however, these patterns were only present in a few cases and were associated with DAD. More advanced and fibrotic interstitial changes were observed in cases with prolonged COVID-19, forming “pseudo-cysts,” corresponding to severe interstitial fibrosis due to excessive deposition of extracellular matrix, in some cases associated with barotrauma.

Pulmonary microthrombosis is prevalent in our series and is already considered as characteristic of COVID-19. We showed previously that lung microthrombosis has no association with the time of evolution, being present in most severe cases already in the first days of admission to the ICU [20]. In a previous report, we described an initial 80% incidence of microthrombosis in 10 patients [14, 15]. This finding had an important impact on the understanding of COVID-19 pathogenesis over time and on the management of critically ill patients to prevent pulmonary and systemic thrombosis [32]. In the present study, the prevalence of pulmonary microthrombi was lower (63.3%) than previously described by our group and lower than found in other case series (71.8%) [28]; this difference may be either because of changes in treatment protocols [32], or related to variability on representativeness of a multifocal and sparse event. When comparing with CDA studies, the rate of pulmonary thrombosis is similar (73% in a recent review) [33]. The small difference in the incidence might be explained by the identification of thrombosis in larger vessels in CDAs, which is not ideally evaluated by MITS.

Secondary suppurative bacterial pneumonia was found in more than half of our patients, a rate in agreement with other studies [21]. Invasive pulmonary aspergillosis has been described in COVID-19, associated with prolonged mechanical ventilation, severe lymphopenia and immune dysregulation, and the use of corticosteroids and broad-spectrum antibiotics [34]. In our series, we found only 1 case of pulmonary invasive fungal infection, caused by *Candida* spp. Coinfection of COVID-19 pneumonia and miliary tuberculosis was observed in 1 case, which could be an expected association in a country with high incidence of mycobacterial infections.

The IHC reaction in the lung is positive in the pulmonary alveolar and ciliary epithelium, in a granular cytoplasmic pattern. The IHC positivity can significantly decrease or be negative in cases with prolonged disease (≥20 days), as reported by others [35]. Although we did not perform IHC in all cases, these time-related changes in positivity were also observed in our cases.

In extrapulmonary organs, the histological alterations were related to SARS-CoV-2 infection/acute inflammatory systemic disease or to previous chronic diseases [21, 22, 36]. Microthrombi were observed mainly in glomerular tufts and in cerebral and cardiac vessels, corroborating the diagnosis of disseminated intravascular coagulation in COVID-19 [30–34].

Despite a suggestive clinical presentation, 30 patients (37.5%) did not have SARS-CoV-2 identified during their lifetime. The autopsy allowed the diagnostic confirmation of these cases, with the use of several methods (postmortem NP-PCR, tissue RT-PCR, IHC, or EM). In 4 of these patients, both pre- and postmortem NP-PCR were negative, and the final diagnosis was only possible with the molecular analysis of lung tissue collected at US-guided MITS. This result shows the ability of US-guided MITS to confirm the diagnosis of COVID-19 by different ancillary methods, which is important for epidemiological records during the pandemic.

US-guided MITS also allowed us to assess the invasive potential of SARS-CoV-2, with the detection of the virus in various tissues, such as the heart [17, 37], intestine and brain [17], periodontal tissue [38], and major and minor salivary glands [39]. These findings have an impact both on the identification of possible viral reservoirs and on understanding the pathophysiology of distinct clinical manifestations.

Five patients were children or adolescents and had a distinct clinical–pathological presentation. In the context of a scarcity of studies of severe COVID-19 in children, we were able to study a particular aspect of the disease, that is, the MIS-C, with new information on the mechanisms involved in the pathogenesis of the syndrome [17, 37].

US was useful not only as a guide for biopsies but also for assessing the different phases and extent of SARS-CoV-2 pneumonia. The correlation of the US image with the pulmonary histological alterations demonstrated that lung US can be used in the follow-up of critically ill COVID-19 patients for the early detection of pulmonary remodeling in patients on mechanical ventilation [13, 20].

After years of experience with the US-guided MITS, recent modifications in the method show that it is possible to expand tissue sampling, overcoming the possible limitations of small biopsies. The use of the scalpel to dissect the lungs, heart, and intestine allows us to obtain large amounts of tissue using small openings in the skin, preserving the concept of minimal invasiveness. These adaptations might improve the study of focal or heterogeneous diseases or the evaluation of sites hitherto not accessed by the US-guided MITS, such as the large pulmonary and cardiac vessels, expanding the perspectives for the use of MITS.

The application of US-guided MITS allowed us to provide critical responses for the attending physicians and to investigate the pathogenesis of a new infectious agent such as SARS-CoV-2 in consonance with the literature [40]. None of the personnel involved in the procedure were contaminated during the period of this study. This is consistent with other recent reports on biosafety of US-guided MITS [27]. We also took advantage of its portability, performing the autopsies inside the hospital in some cases, which reduced the body release time.

The limitations of this study include the use of PCR and IHC only in selected samples to detect SARS-CoV-2 in tissues. On the other hand, this is, to our knowledge, the largest series of MITS performed in COVID-19 patients, including different ages and time courses of the disease, which allowed a detailed characterization of pathological findings and evaluation of the accuracy of the method.

COVID-19 seems to have taken autopsies out of the limbo where they have been for decades. During the pandemic, autopsies provided important information about the damage patterns of the disease, opening venues for several studies that dissect the pathogenic pathways of SARS-CoV-2. Our data showed that the US-guided MITS approach is safe, reproducible, and has the capacity similar to a CDA to build knowledge to help fight this terrible and still indomitable disease.

## Supplementary Data

Supplementary materials are available at *Clinical Infectious Diseases* online. Consisting of data provided by the authors to benefit the reader, the posted materials are not copyedited and are the sole responsibility of the authors, so questions or comments should be addressed to the corresponding author.

ciab885_suppl_Supplementary_Materials

## Notes

***Acknowledgments.*** The authors acknowledge all healthcare providers involved in the care of the patients with coronavirus disease 2019 (COVID-19); all hospital (Clinics Hospital, University of São Paulo School of Medicine) and São Paulo Autopsy Service staff who have taken part in the Coronavirus Crisis Task Force during the epidemic season; and the Núcleo de Vigilância Epidemiológica for providing us hospital data during the epidemic period. The authors also acknowledge and are deeply thankful to all relatives and legal representatives who consented to the postmortem examinations of their beloved relatives lost to COVID-19.

***Author contributions.*** E. P. O. was involved in the care of the patients. T. L. L. F. L. and C. S. M. interviewed the next of kin. A. N. D. N., R. M., J. T. F., and P. H. N. S. collected autopsy samples and ultrasound data. A. N. D. N., F. C. G. A., L. F. F. S., M. D., and T. M. interpreted the autopsy morphological and clinical data. M. S. G. G. and J. R. R. P. performed and interpreted the molecular data. K. C. S. B., C. A., and A. B. S. prepared the slides. C. T. K. performed immunohistochemistry. E. G. C. performed and interpreted the electron microscopy analysis. L. F. F. S., A. N. D. N., M. D., T. M., and P. H. N. S. prepared the original draft of the manuscript. All authors reviewed and approved the final version of the manuscript.

***Supplement sponsorship.*** This supplement is sponsored by MITS Surveillance Alliance Secretariat, led by RTI International, with funding from the Bill and Melinda Gates Foundation.

***Financial support.*** This work was supported by the Bill & Melinda Gates Foundation (grant number INV-002396); Conselho Nacional de Desenvolvimento Científico e Tecnológico (grant numbers 401825/2020-5 and 304987/2017-4 to M. D. and 304277/2019-3 to T. M.); Fundação de Amparo à Pesquisa do Estado de São Paulo (grant number 2013/17159-2); and Hospital das Clinicas da Faculdade de Medicina da Universidade de Paulo–HC Convida (grant number HC-024-2020). C. S. d. M. reports support from the Brazilian National Council for Scientific and Technological Development.

***Potential conflicts of interest.*** All authors: No reported conflicts of interest. All authors have submitted the ICMJE Form for Disclosure of Potential Conflicts of Interest.

## References

[1] Ferigato S, Fernandez M, Amorim M, Ambrogi I, Fernandes LMM, Pacheco R. The Brazilian government’s mistakes in responding to the COVID-19 pandemic. Lancet 2020; 396:1636. 10.1016/S0140-6736(20)32164-4 PMC7575269 33096042

[2] World Health Organization. Coronavirus disease 2019 (COVID-19) data. 2021. Available at: https://covid19.who.int/region/amro/country/br. Accessed 22 March 2021.

[3] Castillo P, Martínez MJ, Ussene E, et al. Validity of a minimally invasive autopsy for cause of death determination in adults in Mozambique: an observational study. PLoS Med 2016; 13:e1002171. 27875530 10.1371/journal.pmed.1002171 PMC5119723

[4] Wong EB, Omar T, Setlhako GJ, et al. Causes of death on antiretroviral therapy: a post-mortem study from South Africa. PLoS One 2012; 7:e47542. 23094059 10.1371/journal.pone.0047542 PMC3472995

[5] Cox JA, Lukande RL, Kalungi S, et al. Practice of percutaneous needle autopsy; a descriptive study reporting experiences from Uganda. BMC Clin Pathol 2014; 14:44. 25506261 10.1186/1472-6890-14-44 PMC4265453

[6] Cox JA, Lukande RL, Kalungi S, et al. Needle autopsy to establish the cause of death in HIV-infected hospitalized adults in Uganda: a comparison to complete autopsy. J Acquir Immune Defic Syndr 2014; 67:169–76. 25072614 10.1097/QAI.0000000000000290

[7] Castillo P, Ussene E, Ismail MR, et al. Pathological methods applied to the investigation of causes of death in developing countries: minimally invasive autopsy approach. PLoS One 2015; 10:e0132057. 26126191 10.1371/journal.pone.0132057 PMC4488344

[8] Duarte-Neto AN, Monteiro RAA, Johnsson J, et al. Ultrasound-guided minimally invasive autopsy as a tool for rapid post-mortem diagnosis in the 2018 Sao Paulo yellow fever epidemic: correlation with conventional autopsy. PLoS Negl Trop Dis 2019; 13:e0007625. 31329590 10.1371/journal.pntd.0007625 PMC6675127

[9] Soper FL. Yellow fever: the present situation (October 1938) with special reference to South America. Trans R Soc Trop Med Hyg 1938; 32:297–322.

[10] Soper FL. Boletin de la Oficina Sanitaria Panamericana (OSP): Febre Amarela Panamericana, 1938 a 1942. Rev Panam Salud Publica 1942; 21:1207–22.

[11] Mauad T, Nascimento FBPD, Dolhnikoff M, Picka MCM, Saldiva PHN; BIAS. Pulmonary interstitial emphysema in fatal asthma: case report and histopathological review. BMC Pulm Med 2018; 18:50. 29554886 10.1186/s12890-018-0615-7 PMC5859394

[12] Centers for Disease Control and Prevention. Collection and submission of postmortem specimens from deceased persons with confirmed or suspected COVID-19. 2020. Available at: https://www.cdc.gov/coronavirus/2019-ncov/hcp/guidance-postmortem-specimens.html. Accessed 22 March 2021.

[13] de Almeida Monteiro RA, Duarte-Neto AN, Ferraz da Silva LF, et al. Ultrasound assessment of pulmonary fibroproliferative changes in severe COVID-19: a quantitative correlation study with histopathological findings. Intensive Care Med 2021; 47:199–207. 33392642 10.1007/s00134-020-06328-4 PMC7779089

[14] Duarte-Neto AN, Monteiro RAA, da Silva LFF, et al. Pulmonary and systemic involvement in COVID-19 patients assessed with ultrasound-guided minimally invasive autopsy. Histopathology 2020; 77:186–97. 32443177 10.1111/his.14160 PMC7280721

[15] Dolhnikoff M, Duarte-Neto AN, de Almeida Monteiro RA, et al. Pathological evidence of pulmonary thrombotic phenomena in severe COVID-19. J Thromb Haemost 2020; 18:1517–9. 32294295 10.1111/jth.14844 PMC7262093

[16] Centers for Disease Control and Prevention. Coronavirus disease 2019 (COVID-19). Interim guidelines for collecting, handling, and testing clinical specimens from persons for coronavirus disease 2019 (COVID-19). 2021. Available at: https://www.cdc.gov/coronavirus/2019-ncov/lab/guidelines-clinical-specimens.html. Accessed 25 February 2021.

[17] Duarte-Neto AN, Caldini EG, Gomes-Gouvêa MS, et al. An autopsy study of the spectrum of severe COVID-19 in children: from SARS to different phenotypes of MIS-C. EClinicalMedicine 2021; 35:100850. 33937731 10.1016/j.eclinm.2021.100850 PMC8072136

[18] World Health Organization; Corman VM, Bleicker T, Brunink S, et al. Diagnostic detection of 2019-nCoV by real-time RT-PCR. 2020. Available at: https://www.who.int/docs/default-source/coronaviruse/protocol-v2-1.pdf. Accessed 2 April 2020.

[19] Corman VM, Landt O, Kaiser M, et al. Detection of 2019 novel coronavirus (2019-nCoV) by real-time RT-PCR. Euro Surveill 2020; 25:2000045. 10.2807/1560-7917.ES.2020.25.3.2000045 PMC6988269 31992387

[20] Mauad T, Duarte-Neto AN, da Silva LFF, et al. Tracking the time course of pathological patterns of lung injury in severe COVID-19. Respir Res 2021; 22:32. 33514373 10.1186/s12931-021-01628-9 PMC7844838

[21] Menter T, Haslbauer JD, Nienhold R, et al. Postmortem examination of COVID-19 patients reveals diffuse alveolar damage with severe capillary congestion and variegated findings in lungs and other organs suggesting vascular dysfunction. Histopathology 2020; 77:198–209. 32364264 10.1111/his.14134 PMC7496150

[22] Hooper JE, Padera RF, Dolhnikoff M, et al. A postmortem portrait of the coronavirus disease 2019 (COVID-19) pandemic: a large multi-institutional autopsy survey study. Arch Pathol Lab Med 2021; 145:529–35. 33449998 10.5858/arpa.2020-0786-SA

[23] Bryce C, Grimes Z, Pujadas E, et al. Pathophysiology of SARS-CoV-2: the Mount Sinai COVID-19 autopsy experience. Mod Pathol 2021; 34:1456–67. 33795830 10.1038/s41379-021-00793-y PMC8015313

[24] Maiese A, Manetti AC, La Russa R, et al. Autopsy findings in COVID-19-related deaths: a literature review. Forensic Sci Med Pathol 2021; 17:279–96. 33026628 10.1007/s12024-020-00310-8 PMC7538370

[25] Vasquez-Bonilla WO, Orozco R, Argueta V, et al. A review of the main histopathological findings in coronavirus disease 2019. Hum Pathol 2020; 105:74–83. 32750378 10.1016/j.humpath.2020.07.023 PMC7395947

[26] Tian S, Xiong Y, Liu H, et al. Pathological study of the 2019 novel coronavirus disease (COVID-19) through postmortem core biopsies. Mod Pathol 2020; 33:1007–14. 32291399 10.1038/s41379-020-0536-x PMC7156231

[27] Rakislova N, Marimon L, Ismail MR, et al. Minimally invasive autopsy practice in COVID-19 cases: biosafety and findings. Pathogens 2021; 10:412. 33915771 10.3390/pathogens10040412 PMC8065952

[28] Flikweert AW, Grootenboers MJJH, Yick DCY, et al. Late histopathologic characteristics of critically ill COVID-19 patients: different phenotypes without evidence of invasive aspergillosis, a case series. J Crit Care 2020; 59:149–55. 32674001 10.1016/j.jcrc.2020.07.002 PMC7340597

[29] Beasley MB, Franks TJ, Galvin JR, Gochuico B, Travis WD. Acute fibrinous and organizing pneumonia: a histological pattern of lung injury and possible variant of diffuse alveolar damage. Arch Pathol Lab Med 2002; 126:1064–70. 12204055 10.5858/2002-126-1064-AFAOP

[30] Copin MC, Parmentier E, Duburcq T, Poissy J, Mathieu D; Lille COVID-19 ICU and Anatomopathology Group. Time to consider histologic pattern of lung injury to treat critically ill patients with COVID-19 infection. Intensive Care Med 2020; 46:1124–6. 32328726 10.1007/s00134-020-06057-8 PMC7178098

[31] Recovery Collaborative Group, Horby P, Lim WS, Emberson JR, et al. Dexamethasone in hospitalized patients with COVID-19. N Engl J Med 2021; 384:693–704. 32678530 10.1056/NEJMoa2021436 PMC7383595

[32] Negri EM, Piloto BM, Morinaga LK, et al. Heparin therapy improving hypoxia in COVID-19 patients—a case series. Front Physiol 2020; 11:573044. 33192569 10.3389/fphys.2020.573044 PMC7604350

[33] Parra-Medina R, Herrera S, Mejia J. Systematic review of microthrombi in COVID-19 autopsies. Acta Haematol 2021; 144:476–83. 33873184 10.1159/000515104 PMC8089413

[34] van Arkel ALE, Rijpstra TA, Belderbos HNA, van Wijngaarden P, Verweij PE, Bentvelsen RG. COVID-19-associated pulmonary aspergillosis. Am J Respir Crit Care Med 2020; 202:132–5. 32396381 10.1164/rccm.202004-1038LE PMC7328331

[35] Martines RB, Ritter JM, Matkovic E, et al;COVID-19 Pathology Working Group. Pathology and pathogenesis of SARS-CoV-2 associated with fatal coronavirus disease, United States. Emerg Infect Dis 2020; 26:2005–15. 32437316 10.3201/eid2609.202095 PMC7454055

[36] Basso C, Leone O, Rizzo S, et al. Pathological features of COVID-19-associated myocardial injury: a multicentre cardiovascular pathology study. Eur Heart J 2020; 41:3827–35. 32968776 10.1093/eurheartj/ehaa664 PMC7543528

[37] Dolhnikoff M, Ferreira Ferranti J, de Almeida Monteiro RA, et al. SARS-CoV-2 in cardiac tissue of a child with COVID-19-related multisystem inflammatory syndrome. Lancet Child Adolesc Health 2020; 4:790–4. 32828177 10.1016/S2352-4642(20)30257-1 PMC7440866

[38] Fernandes Matuck B, Dolhnikoff M, Maia GVA, et al. Periodontal tissues are targets for Sars-Cov-2: a post-mortem study. J Oral Microbiol 2020; 13:1848135. 33391625 10.1080/20002297.2020.1848135 PMC7717160

[39] Matuck BF, Dolhnikoff M, Duarte-Neto AN, et al. Salivary glands are a target for SARS-CoV-2: a source for saliva contamination. J Pathol 2021; 254:239–43. 33834497 10.1002/path.5679 PMC8250228

[40] Paganelli CR, Goco NJ, McClure EM, et al. The evolution of minimally invasive tissue sampling in postmortem examination: a narrative review. Glob Health Action 2020; 13:1792682. 32713325 10.1080/16549716.2020.1792682 PMC7480574

