# Supplementary material for: Ultrasound-Guided Minimally Invasive Tissue Sampling: A Minimally Invasive Autopsy Strategy During the COVID-19 Pandemic in Brazil, 2020
Source: Clin Infect Dis. 2021 Dec 15;73(Suppl 5):S442–53. doi: 10.1093/cid/ciab885 (PMC8672862; doi:10.1093/cid/ciab885)
Supplement: ciab885_suppl_Supplementary_Materials [file ciab885_suppl_supplementary_materials.docx]

Ultrasound-guided minimally invasive tissue sampling: A minimally invasive autopsy strategy during 2020 COVID-19 pandemic in Brazil

Supplementary Information

**Detailed Methods**

**US-guided MITS Protocol**

US-guided MITS was performed using a standard safety protocol in which the deceased bodies were covered with a sealed plastic bag by two trained technicians using the appropriate personal protective equipment, including surgical clothing, impermeable aprons, rubber boots, plastic sleeves, three layers of gloves, a rubber cap, an N95 mask under a surgical mask, and eye protection googles. Access to the autopsy room was limited to two people: the US examiner and the supporting technician. The personnel involved directly or indirectly with the MITS procedure were tested weekly for SARS-CoV-2 with NP-PCR.

We used portable SonoSite M-Turbo R (Fujifilm, Bothell, WA, USA) US equipment with C60x (5–2 MHz Convex) multifrequency broadband transducers and generation of Digital Imaging and Communications in Medicine (DICOM) standard images. The images obtained by US were used to localize and orient the sampling in several organs and to select the most affected areas within each organ. Tissue sampling was performed with Tru-Cut semi-automatic coaxial needles (14G; 20 cm in length). Figure 1 shows representative US images from different organs.

The following tissue samples were collected under US guidance: lungs (eight samples from each lung for histology, including anterior and posterior samples from apical and basal regions of each lung and two samples for molecular analysis), liver (at least two samples), both kidneys (one sample each), spleen (one sample), and heart (one to three samples). Other tissues were sampled without direct image guidance: skeletal muscle (femoral quadriceps), skin (in the left thigh, with a 5-mm punch needle), and brain (trans-sphenoidal puncture). Intestinal tissue, periodontal tissue, parotid and other salivary glands, adipose tissue, bone marrow, and thyroid were collected in selected cases. Tissue samples for molecular tests were included in Falcon Tubes and immediately frozen and stored at −80°C. Tissue samples for histopathological analysis were fixed in buffered 10% formalin, embedded in paraffin, and stained with hematoxylin and eosin and with additional histochemical and immunohistochemical staining when needed. In selected cases, tissue samples were fixed in glutaraldehyde for ultrastructural analyses.

**Molecular and immunohistochemical detection of SARS-CoV-2**

Immunohistochemistry (IHC) to evaluate the distribution of the viral antigen within different morphological pulmonary alterations was performed on lung samples from selected cases to detect SARS-CoV-2 Nucleocapsid protein. The criteria to select cases for IHC analysis included one of the following: cases with negative pre-mortem and post-mortem PCR for SARS-CoV-2 and suggestive pulmonary histology; unusual cases (e.g., children, puerperal women); and cases with particular pulmonary histological findings (e.g., squamous metaplasia, pulmonary “pseudo-cystic” formations), A detailed description of the IHC analysis has been previously reported. The primary antibody was a mouse monoclonal antibody ([6H3] -GeneTex Inc., Irvine, CA, USA), at 1:500 dilution. The antigen retrieval was performed with 10 mM citrate buffer at pH 6.0. Amplification was achieved by alkaline phosphatase conjugated polymer (Polink-2 AP, GBI Labs cat.D24-110, Bothell, Washington, USA), revealed by permanent Fast Red chromogen (GBI-Permanent Red Substrate, GBI Labs cat. C13-120, Bothell, Washington, EUA). To ensure the specificity of the antibody and to avoid false positivity caused by cross reaction with other respiratory viruses, the primary antibody was tested in lung samples from patients who died before the COVID-19 pandemic from Influenza H1N1 pneumonia, measles, syncytial respiratory virus, herpes virus, cytomegalovirus, and adenovirus. The IHC staining was negative in all these samples.

We employed PCR for RNA detection of SARS-CoV-2 in frozen lung tissue of all patients that did not have the diagnosis confirmation prior to death. Tissues were stored at −80°C and submitted to homogenization using the FastPrep™ instrument (MP Biomedical; São Caetano do Sul, SP, Brazil), and nucleic acid extraction was performed using the TRIzol® reagent (Invitrogen, Carlsbad, CA, USA). Molecular detection of SARS-CoV-2 was performed using SuperScript™ III PlatinumTM One-Step qRT-PCR Kit (Invitrogen) with primers and probes described in the Centers for Disease Control and Prevention and Charité protocols that amplify the region of the nucleocapsid N gene (2019_nCoV_N1 assay) and E gene (E_Sarbeco assay), respectively. Human RNase P gene was also amplified as nucleic acid extraction control. The reactions were carried out in a 7500 Fast Real-Time PCR System (Applied Biosystems, Foster City, CA, USA) and consisted of a reverse transcription at 50°C for 15 min, followed by incubation at 95°C for 2 min, and 45 cycles of temperature varying from 95°C for 15 s to 55°C (N and RNAse P genes)/58°C (E gene) for 30s

.

**Ultrastructural Analyses**

Pulmonary tissue samples from seven patients were examined under transmission electron microscope (EM). Tissues were fixed in 2% glutaraldehyde, post-fixed in 1% OsO_4_, stained in 1% aqueous uranyl acetate, and embedded in epoxy resin. Ultrathin sections were double-stained by uranyl acetate and lead citrate. Micrographs were obtained with a Jeol JEM 1010 EM (80 kV; Tokyo, Japan).

**Diagnoses of causes of death by US-guided MITS**

To determine the immediate and underlying cause of death for the enrolled cases, a senior pathologist and an infectious disease specialist considered the following information: (1) patient records, clinical information obtained with the next of kin, and hospital charts; (2) post-mortem ultrasound information; (3) histopathological analysis of all sampled tissues; (4) pre- or post-mortem NP-PCR results when available; (5) lung tissue PCR results when available; (6) SARS-CoV-2 IHC results when available; and (7) ultrastructural analysis when available. When there was any doubt about the final diagnosis, the case was discussed in a panel with three senior pathologists. All reported causes were coded by the São Paulo State Health Bureau according to the International Classification of Diseases, 11th edition.

The references were already listed in the main manuscript
